# Recruiting schools, adolescents and parents to a sexual-health trial: experiences, challenges and lessons learned from the Jack Trial (NCT02092480)

**DOI:** 10.1186/1745-6215-16-S2-P81

**Published:** 2015-11-16

**Authors:** Aine Aventin, Lisa Maguire, Mike Clarke, Maria Lohan

**Affiliations:** 1 Queen's University Belfast, Northern Ireland, UK

## Background

Recruitment to school-based randomised trials is challenging; even more so when the focus of the study is a sensitive issue such as sexual health. The Jack Feasibility Trial aims to determine the facilitators and barriers to recruitment and retention to a school-based sexual health trial and identify optimal multi-level strategies for a full trial.

## Method

The Jack Trial is an NIHR-funded feasibility study of a film-based sexual health intervention, recruiting over 800 adolescents from 8 post-primary schools in Northern Ireland. In order to examine the feasibility of piloted recruitment and retention methods and assess acceptability of participation across the range of schools and individuals approached, we analysed qualitative data from triangulated sources including a parents’ survey and semi-structured interviews with principals, vice-principals, teachers and parents recruited to the study.

## Results

With reference to Social Learning Theory, we identified a number of individual, behavioural and environmental level factors which influenced recruitment and retention. Commonly identified facilitators included the perceived relevance and potential benefit of the intervention to adolescents, the credibility of the organisation running the study, support offered by trial staff, and financial incentives. Key barriers were prior commitment to other research, lack of time and resources, and perceptions that the intervention was incompatible with adolescent needs or school ethos.

## Conclusion

This study highlights pertinent general and trial-specific facilitators and barriers to recruitment to a sexual health trial in a school setting, which will prove useful for successful conduct of future trials with schools, adolescents and parents.

